# COVID19 pneumonia with cavitation and cystic lung changes: multi-detector computed tomography spectrum of a gamut of etiologies

**DOI:** 10.1259/bjro.20210007

**Published:** 2021-07-29

**Authors:** Arunima Aggarwal, Anupama Tandon, Shuchi Bhatt, Anivita Aggarwal, Saloni Dagar, Harshit Bansal

**Affiliations:** 1 Department of Radio-diagnosis, University College of Medical Sciences and GTB Hospital (University of Delhi) Dilshad Graden, New Delhi, India; 2 Department of Medicine, All India Institute of Medical Sciences (AIIMS), Ansari Nagar, New Delhi, India

## Abstract

The COVID19 pandemic since its beginning in March 2020, continues to wreak havoc causing great morbidity and mortality with each passing day. Ample literature is now available describing the imaging features of COVID19 infection; however, there is still a paucity of knowledge on the various causes of pulmonary cavitation and cystic lesions which can be associated with the virus albeit uncommonly. Cavitation in a COVID19 positive patient could be a consequence of the infection itself or a manifestation of sinister etiologies like coinfection with bacterial, fungal or mycobacterial pathogens, or incidental malignancy/metastasis. It could also be a result of multiple cavitating pulmonary nodules as a manifestation of septic emboli and infarct, Granulomatosis with polyangiitis or rheumatoid arthritis creating a diagnostic dilemma. Similarly, the causes of cystic air spaces on chest CT in COVID19 patient can be varied, either primarily due to the infection itself or secondary to coexistent cystic bronchiectasis, emphysema, interstitial lung disease or mechanical ventilation-associated barotrauma as well as complicated pulmonary cysts. Through this pictorial review, we aim to highlight these uncommon imaging manifestations of COVID19 and educate the reader regarding the various causes, MDCT features and differentials to be considered while approaching a cavity/cystic lesion amidst this pandemic.

## Introduction

The novel Coronavirus disease, also known as COVID19 infection first reported in the late 2019, has rapidly spread across the globe and resulted in significant mortality and morbidity.

Because of the pulmonary tropism of the virus, pulmonary manifestations are often encountered in symptomatic patients. Chest CT plays a critical role in the evaluation of COVID19 infection.

The typical CT features include multifocal bilateral rounded ground glass opacities with or without consolidations predominantly in a peripheral and basal distribution with superimposed interlobular septal thickening resulting in a *crazy paving* pattern. These can resolve/heal with varying degree of fibrosis.^1^ Discrete pulmonary nodules, lymphadenopathy, pleural effusion and cavitation have been rarely described. Cavitation and cystic changes have been reported in only about 0.7% of the cases on imaging.^2^ Another rare manifestation is the presence of cystic air spaces (tiny air spaces distinct from cavitation), reported in only a few cases.

Cavities and cystic lesions on chest CT in COVID19 infection can occur due to a gamut of etiologies; either primarily due to COVID19 infection or secondary to various coexisting pathologies (Table 1). Though scattered reports are available, to the best of our knowledge, there is no comprehensive article compiling the various causes. The authors encountered 32 COVID19 cases with cysts and/or cavitation on chest CT in a dedicated COVID hospital. This pictorial review encompasses the CT spectrum of the various cavitatory and cystic lesions which can be seen to develop, coexist or be complicated by COVID19 infection.

**Table 1.** Various causes for cavitation and cystic lesions in COVID19 infection

CAVITATORY LESIONS:	CYSTIC LESIONS:
1. Cavitation due to COVID19 infection	1. Cystic air spaces associated with COVID19
2. Coexistent COVID19 infection with other causes of cavitation-	2. Cystic lung lesions with coexistent COVID19 infection-
a. Bacterial coinfection Non-Mycobacterial infections *Staphylococcus*, *Klebsiella* and *Streptococcus*, etc *Mycobacterium tuberculosis* coinfection- Active, Reactivation and Sequelae of previous	a. Pneumothorax: mechanical ventilation associated barotrauma, spontaneous
	b. Cystic bronchiectasis: Sequelae of COVID19 Sequelae of *Mycobacterium tuberculosis* Kartagener syndrome Sequelae of previous infection
b. Fungal coinfection	c. Emphysematous bullae
c. Septic emboli or pulmonary infarct	d. Simple cyst or Pneumatocoele
d. Neoplastic etiology Primary lung cancer Metastasis Secondary cavitation - post treatment	e. Honeycombing - usual interstitial pneumonia
e. Miscellaneous: Rheumatoid nodules Granulomatosis with polyangiitis	f. Complicated cysts: Hydatid cyst Intraparenchymal bronchogenic cyst

### COVID19-related cavitation and cystic air spaces

#### Cavitation due to COVID19 infection

Lung cavitation, though infrequently seen in viral pneumonias, has been reported in COVID19 infection.^2^ Cavitation has been observed in the absorption stage of disease, usually after 14 days. Although the exact cause remains unknown, predominant histopathological pulmonary finding seen is diffuse alveolar damage. Cavitation can be associated with worsening of symptoms after an initial recovery and a higher morbidity and mortality.

**Figure 1.**
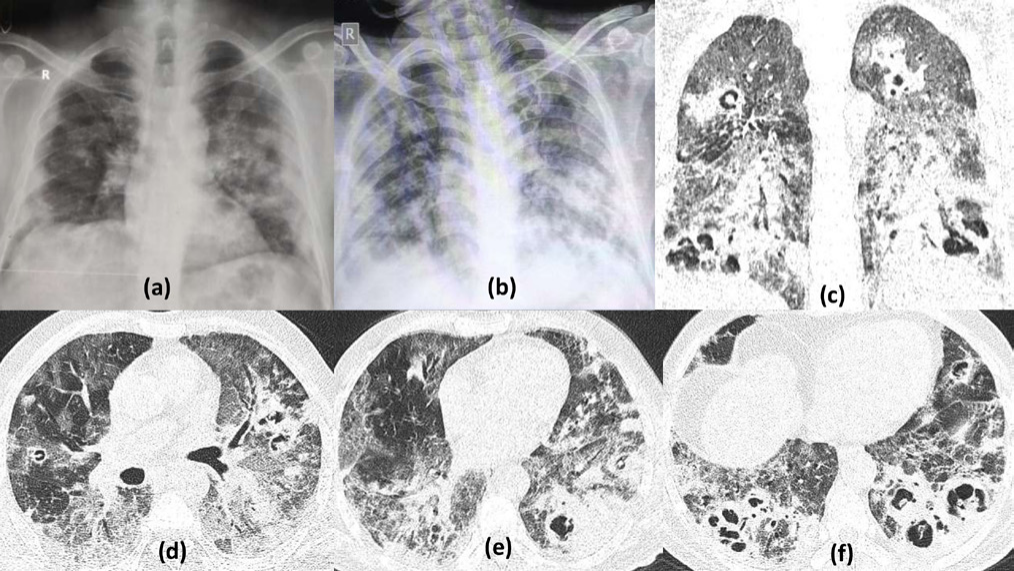
Multiple bilateral cavities due to severe COVID19 pneumonia: 60-year-old male with shortness of breath on high flow oxygen, positive for COVID19 infection. Frontal chest radiograph (a) on Day 6 of illness shows multifocal, fluffy, predominantly peripheral air space opacities in the right lower zone and left mid-lower zone. Serial chest radiograph (b) done on Day 20 (with worsened symptoms) shows progressive increase the infiltrates with a suspicious area of cavitation seen in the left mid zone. HRCT done few days later, axial and coronal sections (c–f) revealed multiple thick-walled cavities of varying sizes having irregular inner walls, within the areas of maximal consolidation bilaterally (predominantly in peripheral and lower lobe distribution typical of COVID19 pneumonitis)

**Figure 2.**
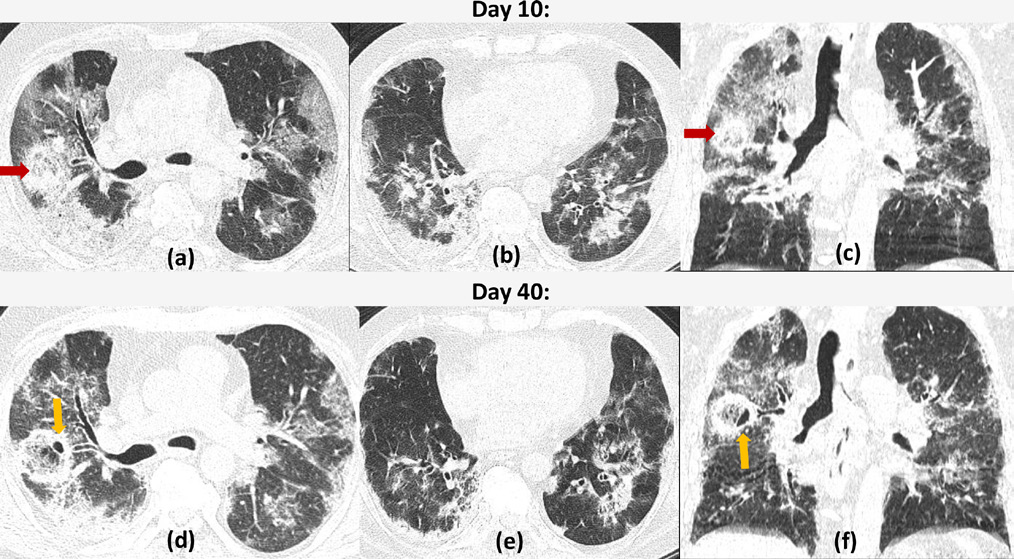
COVID19 with cavitation and *Atoll sign:* A 45-years-old COVID19 positive patient on 10th day of illness. Axial and coronal reformatted CT sections (a–c) show multifocal ground glass opacities and consolidations with a basal and peripheral predominance. A spherical area of central ground glass opacity and peripheral consolidation s/o *Atoll sign* can be seen in the right upper lobe (red arrow). Serial CT (d–f) done 1 month later revealed development of a few small cavitatory lesions (yellow arrows) both in the peripheral rim of consolidation as well as in the central area of ground glass opacities of the right upper lobe lesion. The other peripheral ground glass opacities had slightly reduced and had become more organized.

**Figure 3.**
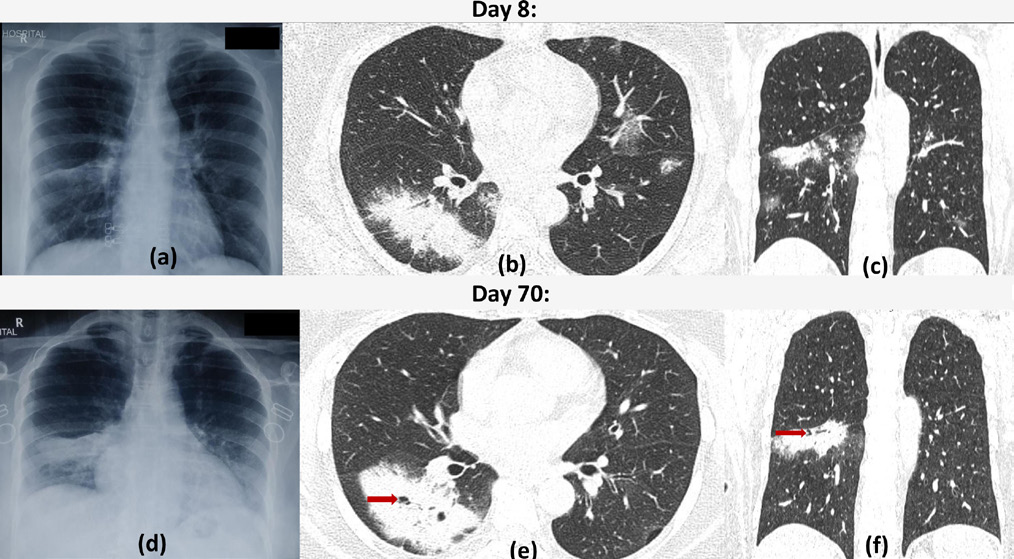
Cavitation within consolidation as a sequelae to COVID19 infection: 52-year-old female with fever tested positive for COVID19 infection, no respiratory complaints. Frontal chest radiograph (a) on the eighth day of illness shows a patchy opacity in the right mid zone. Chest HRCT (axial and coronal lung window sections - b, c) showed a patch of consolidation in the superior basal segment of right lower lobe (atypical feature of COVID19 infection) with bilateral multifocal rounded ground glass opacities. Patients symptoms progressed necessitating high flow oxygen therapy but subsequently improved and was discharged after 10 days. Follow-up frontal radiograph (d) 2 months later shows persistence of the patch. CT sections (e, f) show development of cavitation (red arrows) and non-resolution of consolidation while the ground glass opacities have resolved.

**Figure 4.**
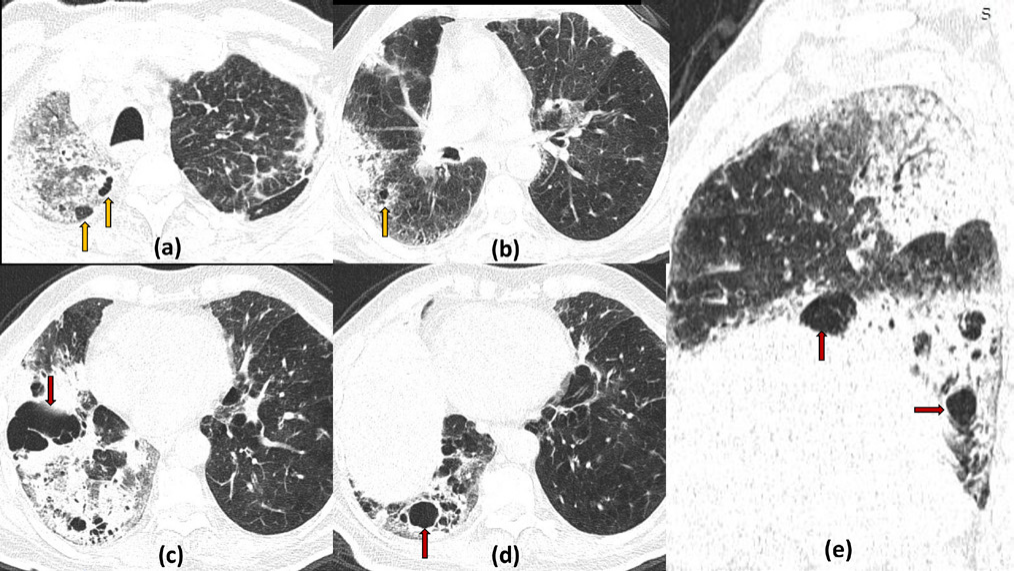
Multiple cavities and cystic air spaces in the absorptive stage of COVID19 infection: 74-year-old COVID19 positive patient on day 26 of illness with persistent high flow oxygen requirement. Axial (a–d) and sagittal (e) lung window sections show patches of consolidation predominantly involving the right lung with multiple variable sized cavities (irregular inner wall and larger size) located within the area of maximal consolidation (red arrow). Also seen are multiple small cystic lesions (smaller with smooth inner wall) in the paraseptal location (yellow arrows). Fibrotic ground glass opacities with interlobular septal thickening with ipsilateral mediastinal shift suggestive of fibrotic sequelae is also present.

On imaging, the cavitatory lesions in COVID19 can be single or multiple, are often variable in size and can be bilateral.^3–6^ They occur in areas of peak disease activity, *i.e*. in regions of previous ground glass opacities or consolidation.^3^ These cavities are thick walled, often have an irregular inner wall and can occasionally have an air fluid level. Signs of fibrosis like interlobular septal thickening, fibrotic ground glass opacities, bronchiectasis or parenchymal bands can be seen in the rest of the lung fields as cavitation occurs in the later stage of the disease^4^ (Figures 1–4).

**Figure 5.**
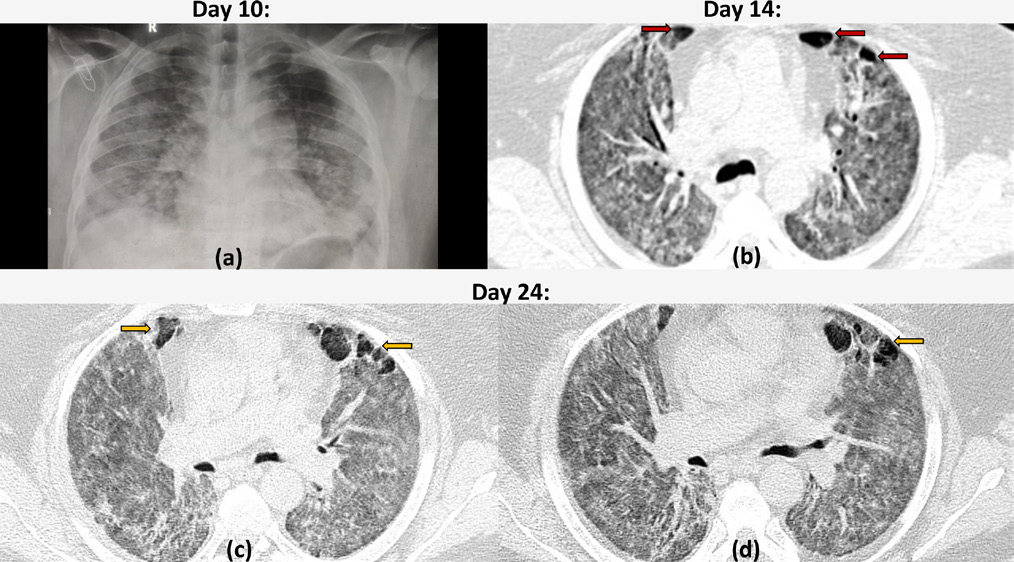
Multiple cystic air spaces in severe COVID19 pneumonia: 53-year-old male with COVID19 infection and severe shortness of breath and low oxygen saturation. Chest radiograph (a) on Day 10, shows multifocal fluffy air space opacities in the bilateral lung fields with an apicobasal gradient. Chest HRCT (axial section a) done on Day 14 of illness shows bilateral diffuse ground glass opacities with interlobular septal thickening. A few cystic air spaces (with regular inner walls) are seen anteriorly in para-septal location (red arrows) (within the middle lobe and lingular segment of left upper lobe) bilaterally. HRCT on Day 24 (c, d) revealed an increase both in number and size of the cystic air spaces (yellow arrows) while the parenchymal changes have become sharper and more organized, although still severe.

Other causes of cavitation like a superadded bacterial, fungal, or mycobacterial infection as well as uncommon causes like a cavitatory infarct, nodule or underlying neoplasm should be excluded by extensive work-up. A comparison with prior imaging can aid in excluding a pre-existing cavity. Serial imaging is mandated to observe any progression or regression of the cavity, and for detection of any complications like pneumothorax.

### Cystic air spaces associated with COVID19

Cystic air spaces are small air containing spaces that have been described in a few published reports in association with COVID19 pneumonia.^7–11^ Their exact pathogenesis remains unclear although damage to alveolar walls by exudates and physiological dilatation of air spaces have been postulated as causative mechanisms.

On chest CT, these appear as multiple small, thin walled air spaces with smooth inner wall, and have been referred to as vacuolar sign,^7^ air bubble sign^9^ or round cystic change.^11^ Preferential locations described are subpleural and/or peribronchovascular.^10^

In our series, seven cases revealed multiple cystic air spaces, subpleural location was seen in four cases while three cases had cysts within the area of pneumonitis or consolidation and no peribronchovascular cysts were observed. Most cases had cysts in the anterior part of the lung (right middle lobe and lingular segment of left lobe), an observation not previously reported. Intralesional cysts have also been mentioned only in a single previous publication^8^ (Figures 4 and 5).

**Figure 6.**
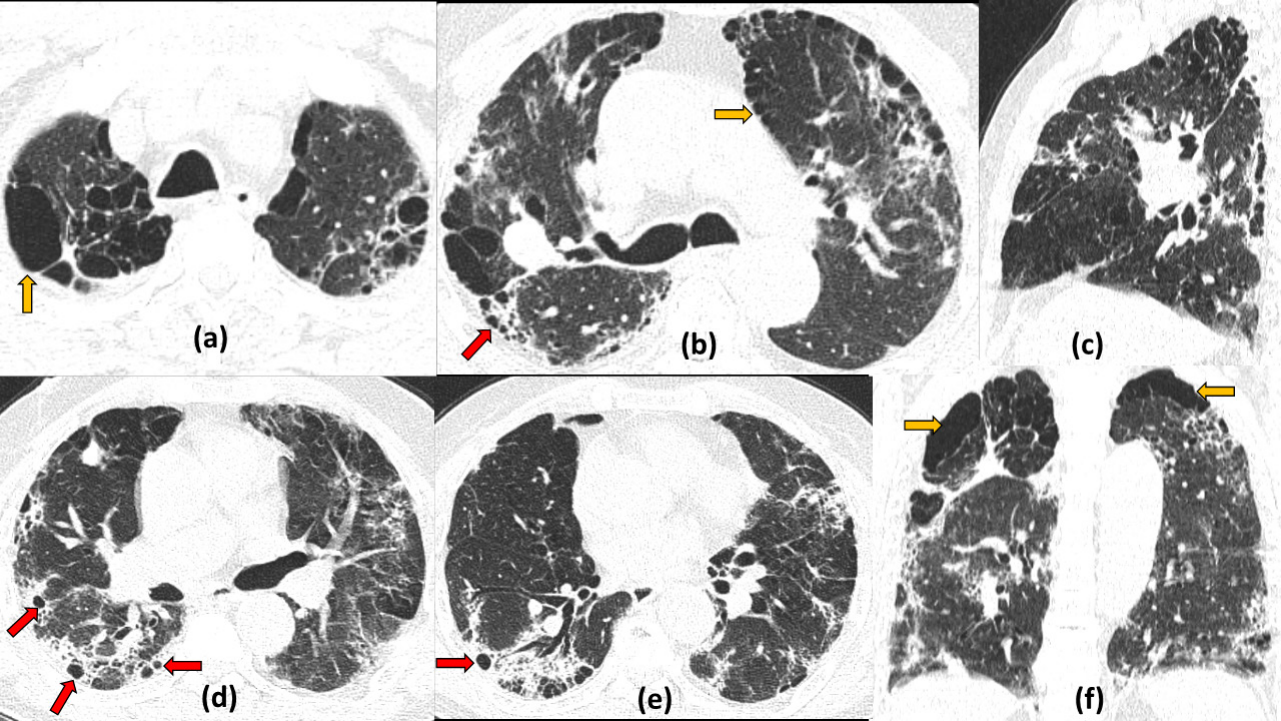
Emphysematous bullae with cystic air spaces as COVID19 sequelae: 67-year-old smoker and a known case of COPD with COVID19 illness. Axial (a, b, d, e), sagittal (c) and coronal (f) CT sections on Day 20 of illness, show multiple tiny relatively thick-walled cystic air spaces (red arrows) noted both in the subpleural and intralesional location, seen along the distribution of the post-COVID19 fibrotic changes in the bilateral peripheral lung fields. In addition, larger thin-walled emphysematous bullae/blebs are seen in the upper sections of the chest (yellow arrows) largely confined to areas uninvolved with the fibrotic changes. These bullae are possibly due to pre-existing COPD (However, no previous HRCT was available for comparison). COPD, chronic obstructive pulmonary disease.

Presence of cysts can increase the specificity of diagnosis as these are not reported with other viral pneumonias although, a differentiation from pre-existing emphysema or interstitial lung disease is essential. Small cystic lesions conforming to areas of prior or current ground glass opacities and consolidations, favor the association with COVID19, while a more random distribution, large and variable size points to other etiologies (Figure 6).

### Cavitation with coexistent COVID19 infection

#### Bacterial coinfections with COVID19 infection

##### Non-mycobacterial infections

Bacterial coinfections as a result of reduced host immunity in COVID19 have been reported in 7% of hospitalized patients and 14% of ICU patients,^12^ and these can adversely impact the prognosis. Commonly isolated bacterial co-pathogens include *Streptococcus pneumoniae, K. pneumoniae, M. pneumoniae, H. influenzae* and *P. aeruginosa*.^12^ *Klebsiella*, *Staphylococcus* and *Streptococcus* are commonly associated with cavitation.

**Figure 7.**
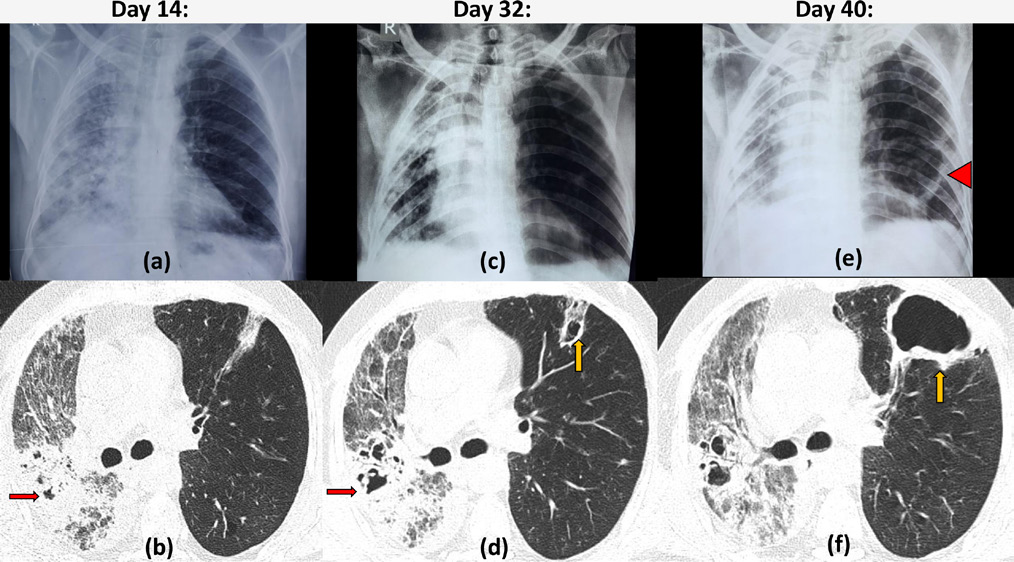
Bacterial coinfection with COVID19 infection: 50-year-old diabetic male with chest tightness and dyspnoea since 10 days, tested positive for COVID19. Imaging on Day 14: frontal chest radiograph (a) revealed marked asymmetrical involvement with infiltrates in the entire right lung and relative sparing of the left lung. HRCT axial section (b) revealed patchy consolidation with few tiny cystic areas within (red arrow). Associated interlobular septal thickening and mild volume loss of right lung was also present. Imaging on Day 32: patient had only slight clinical improvement chest radiograph (c) shows marked ipsilateral mediastinal shift and volume loss with some resolution of infiltrates. HRCT (d) revealed increased size and number of the areas of cavitation which can be seen bilaterally now in areas of previous consolidation (yellow arrow) and were assumed to be a sequelae of COVID19. Imaging on Day 40: patient had clinically worsened Chest radiograph (e) revealed a large cavity in the left lower zone (arrowhead). HRCT (f) revealed marked increase in the size of the cavity in the lingular segment showing thick irregular walls (yellow arrow), disproportionate to the area of previous consolidation, a superinfection was suspected. The features in the right lung remained unchanged. On sputum culture, *Klebsiella pneumoniae* was isolated.

Bacterial coinfections are likely to pose a diagnostic challenge both for the clinician and radiologist. Imaging features atypical for COVID19 like lobar consolidation, pleural effusion, mediastinal lymphadenopathy and cavitation should prompt the radiologist to consider a bacterial coinfection (Figure 7).

##### Mycobacterium tuberculosis coinfections

In an endemic country like India, coinfection with pulmonary tuberculosis (PTB) in a COVID19 patient is commonly seen and its incidence further increases due to a suppressed cellular immunity and high dose corticosteroid administration in moderate to severe COVID19 infection.

**Figure 8.**
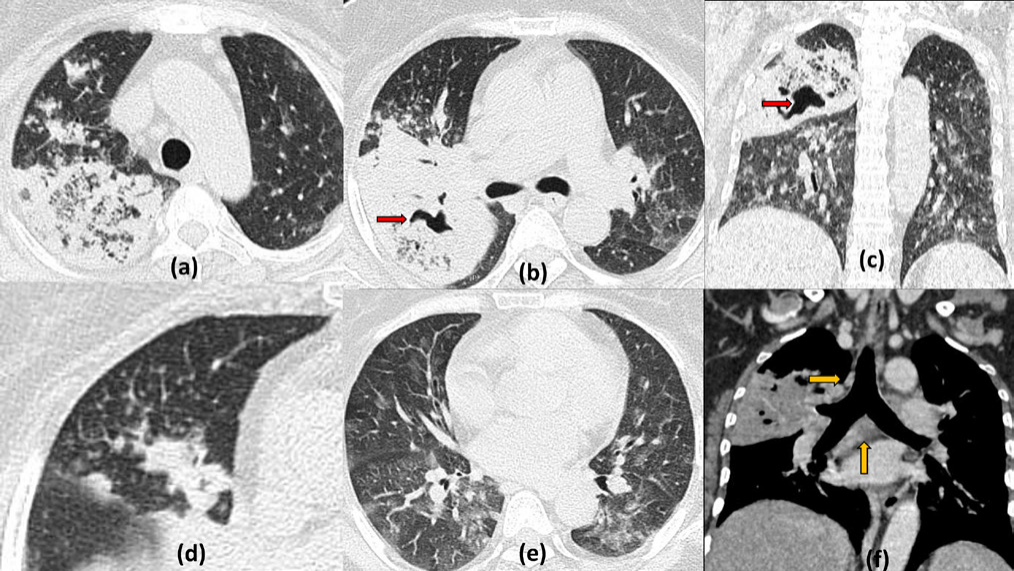
Active cavitating *Mycobacterium tuberculosis* coinfection with COVID19: 46-year-old female presented with high grade fever and acute shortness of breath and tested positive for COVID19 infection. Contrast enhanced CT lung window sections of the chest (a–e) revealed right upper lobe consolidation with central cavitation (red arrow) with multiple centrilobular nodules, some showing tree in bud appearance (zoomed image d). Multifocal rounded ground glass opacities were also seen in the bilateral lower lobes due to Covid19 pneumonitis. Coronal mediastinal window (f) shows heterogeneously enhancing subcarinal and paratracheal mediastinal lymph nodes. Sputum tested positive for Acid Fast Bacillus confirming *Mycobacterial* coinfection.

**Figure 9.**
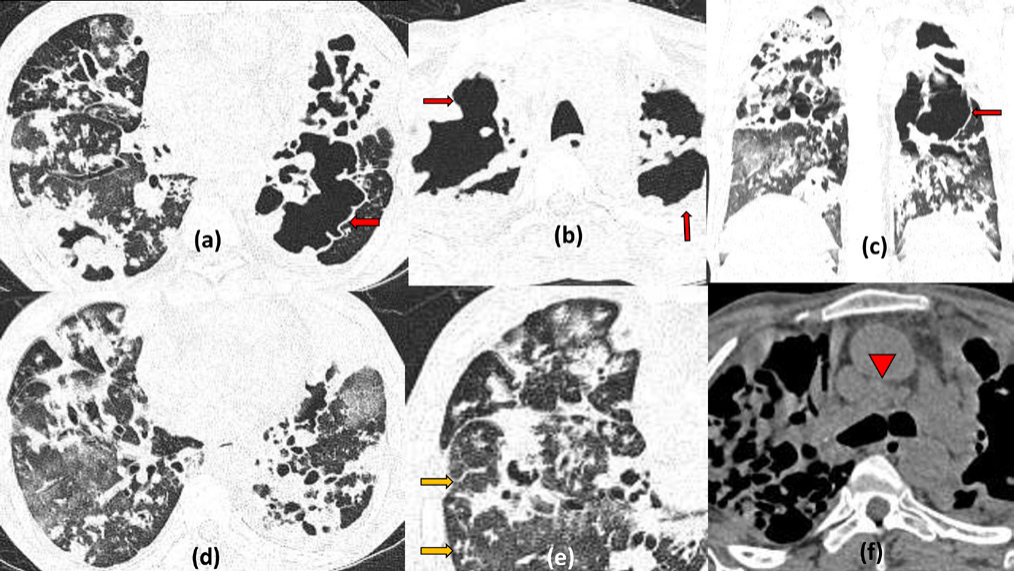
Reactivation *Mycobacterium tuberculosis* coinfection with COVID19 forming multiple cavities: 38-years-old COVID positive male with past history of pulmonary tuberculosis, presented to the emergency with respiratory distress. Chest HRCT axial and coronal lung window sections (a–e) shows multiple variable sized cavities with irregular walls predominantly in upper lobes (red arrows), multifocal patchy consolidation and centrilobular nodules (few showing tree in bud appearance distributed in bilateral lung fields (yellow arrow in zoomed image e). Lower lung fields also show patchy ground glass opacities suggestive of acute COVID19 infection. Axial mediastinal window (non-enhanced - f) shows few subcentimetric pre-carinal lymph nodes (arrowhead). Positive results were obtained of sputum CBNAAT for *Mycobacterial tuberculosis* and RTPCR for SARS-CoV-2 virus confirming coinfection.

**Figure 10.**
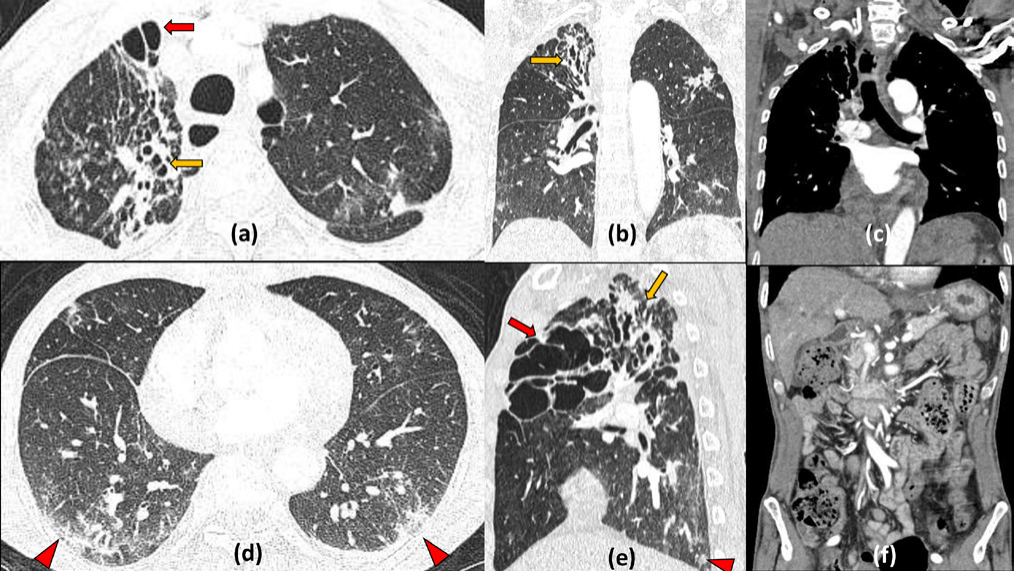
Multiple upper lobe cavities as sequelae of previous *Mycobacterium tuberculosis* with COVID19 infection: 21-year-old male with past history of pulmonary tuberculosis, developed moderate COVID19 pneumonitis. CECT chest axial (a, d), coronal (b) and sagittal (e) sections depict multiple right upper lobe cavities (red arrow) and bronchiectasis (yellow arrow) with ipsilateral mediastinal shift and slight volume loss as a sequelae of previous pulmonary tuberculosis. Multiple patchy peripheral ground glass opacities with interlobular septal thickening are also seen predominantly in the basal lower lobes (arrowheads). Coronal chest and abdomen sections (c, f) reveal no significant abnormality to suggest reactivation of tuberculosis; sputum culture and staining was negative for Acid Fast Bacillus.

The COVID19 patients can either develop primary active tubercular coinfection or superinfection (Figure 8), or there can be a reactivation of previous infection (Figure 9). Also, COVID19 may be coexistent in a patient with sequelae of previous tubercular infection (Figure 10). The imaging findings are summarized in Table 2.

**Table 2.** Types of pulmonary tubercular coinfections with imaging features

Categorization of tubercular coinfection	COVID19 with active pulmonary tubercular coinfection	COVID19 with sequelae of previous pulmonary tuberculosis	COVID19 with reactivation of pulmonary tuberculosis
History of previous pulmonary tuberculosis	Absent	Present	Present
Imaging features of active tuberculosis: Multifocal consolidation with cavitation, usually involving the upper lobes; centrilobular nodules which may show tree-in-bud appearance; necrotic mediastinal lymphadenopathy; pleural effusion	Present	Absent	Present
Imaging features of previous pulmonary tuberculosis: Solitary/multiple cavities predominantly in the upper lobes with associated features of fibrosis: tractional bronchiectasis, volume loss, fibrotic bands, calcified granulomas, calcified mediastinal lymph nodes	Absent	Can be present	Can be present
Imaging features of COVID19 pneumonitis: Multifocal rounded ground glass opacities predominately in periphery and lower lobes, associated with interlobular septal thickening	Can be present	Can be present	Can be present

Tubercular coinfection can result in a higher probability of a severe and critical COVID19 infection, a delayed recovery, and a higher mortality. Thus, a high index of suspicion is essential, and a prompt testing is warranted.

### Fungal coinfection with COVID19 infection

Fungal coinfection with COVID19 infection is on a rise possibly because of immune dysregulation, increasing use of corticosteroids and more awareness amongst clinicians; COVID19-associated invasive pulmonary aspergillosis (CAPA) infection has been observed in as many as 20–35% of severely ill and immunocompromised patients.^13^ Other coexisting fungal lesions were due to subacute pulmonary aspergillosis,^14^ Candida, Mucor or Cryptococcal infections.^13^

**Figure 11.**
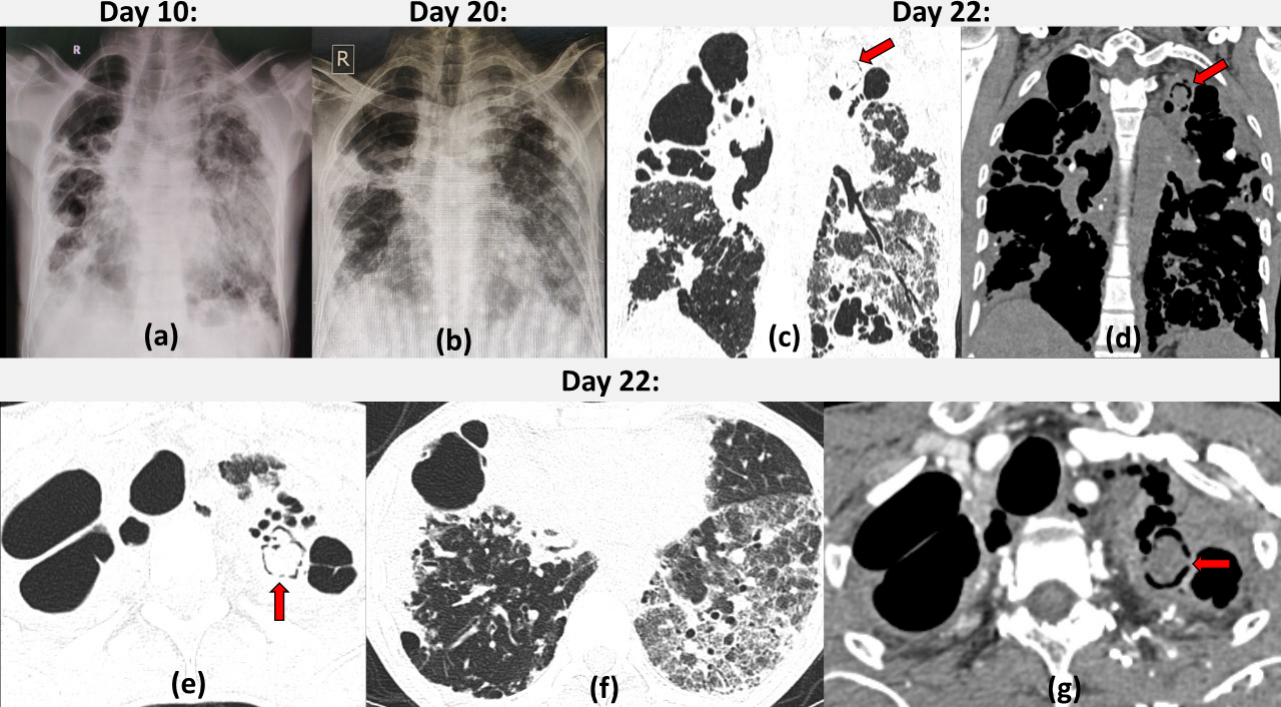
Fungal ball in previous tubercular cavity with COVID19 infection: 50-year-old male with uncontrolled diabetes and past history of pulmonary tuberculosis with low grade fever and productive cough, now tested positive for COVID19. Frontal radiograph (a) on Day 10 of illness shows bilateral fluffy opacities in the left mid zone and bilateral lower zones and multiple variable sized cavities in bilateral upper and right mid-lower zone. Patient became afebrile on Day 15 but had persistent productive cough. Serial chest radiograph (b) on Day 20 reveals marked reduction in bilateral lower zone infiltrates with persistent cavities. CECT chest on Day 22 (c–g): multiple thin-walled variable sized cavities in bilateral upper and right middle lobe. A spherical non-enhancing soft tissue density ball like intracavitatory lesion is seen within a cavity in left apicoposterior segment lined by air peripherally (*air crescent sign*) suggestive of fungal ball (red arrow). Multiple scattered calcific densities are also seen on coronal NCCT image (d). Left lower lobe has fibrotic sequalae of COVID infection.

On HRCT, CAPA usually presents as nodules with a halo sign with cavitation being an uncommon finding, while subacute aspergillosis is seen as a fungal ball within a cavity with an air-crescent sign (Figure 11).

### Cavitation secondary to embolism and infarcts

COVID19 infection predisposes to pulmonary embolism due to its prothrombotic state which can lead to pulmonary infarcts. On Chest CT, infarcts appear as multiple peripheral wedge-shaped opacities which may show internal cavitation,^15^ although uncommon.

**Figure 12.**
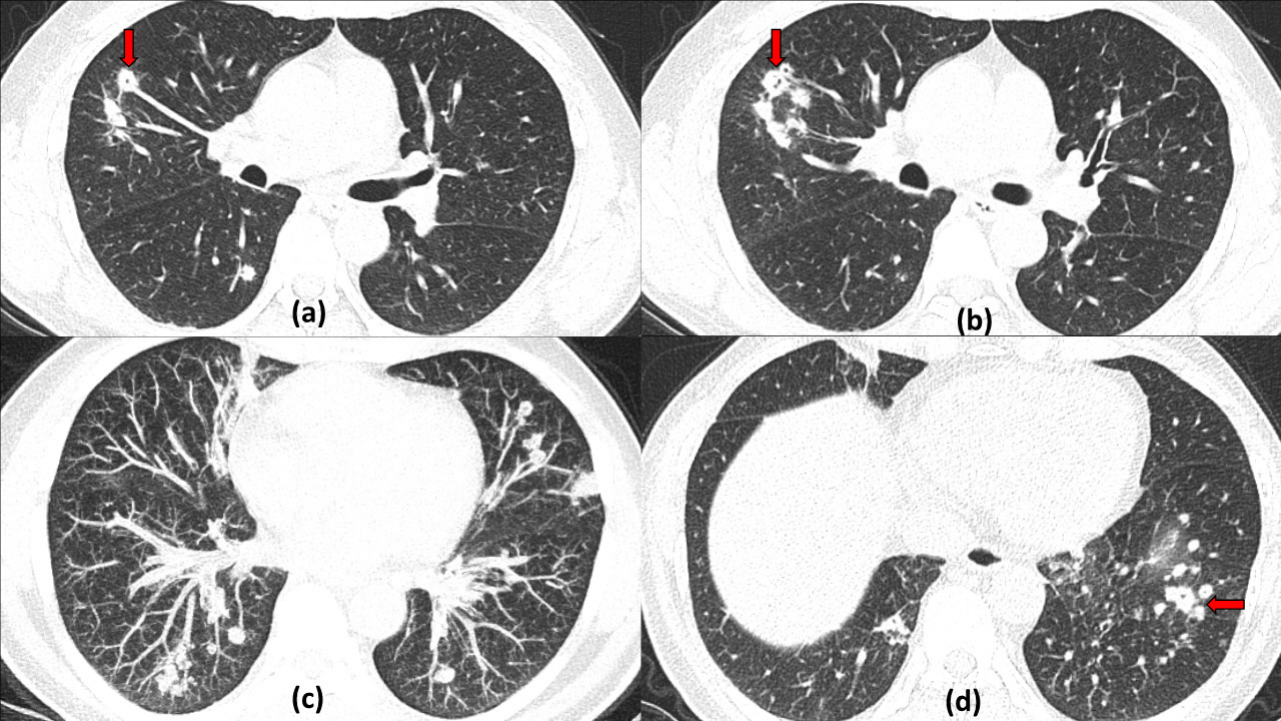
Cavitatory septic emboli with COVID19 infection 46-year-old male diabetic with left leg gangrene presented to surgery emergency and tested positive during routine screening on admission. Axial sections of chest HRCT show randomly scattered multiple well-defined nodules with a few showing central cavitation (red arrows) involving both lungs. At most places, a vessel leading to the nodule suggestive of *feeding vessel sign is* seen (better appreciated on the Maximum Intensity Projection image c). Few areas of focal ground glass opacities were seen in the basal areas of left lung (d) possibly due to COVID pneumonia.

Septic emboli can be seen as multiple nodules distributed peripherally, with majority showing a central cavitation and a *feeding vessel sign*^15^ in a background of COVID19 pneumonitis (Figure 12).

### Cavitation due to neoplastic etiology

#### Primary lung cancer

**Figure 13.**
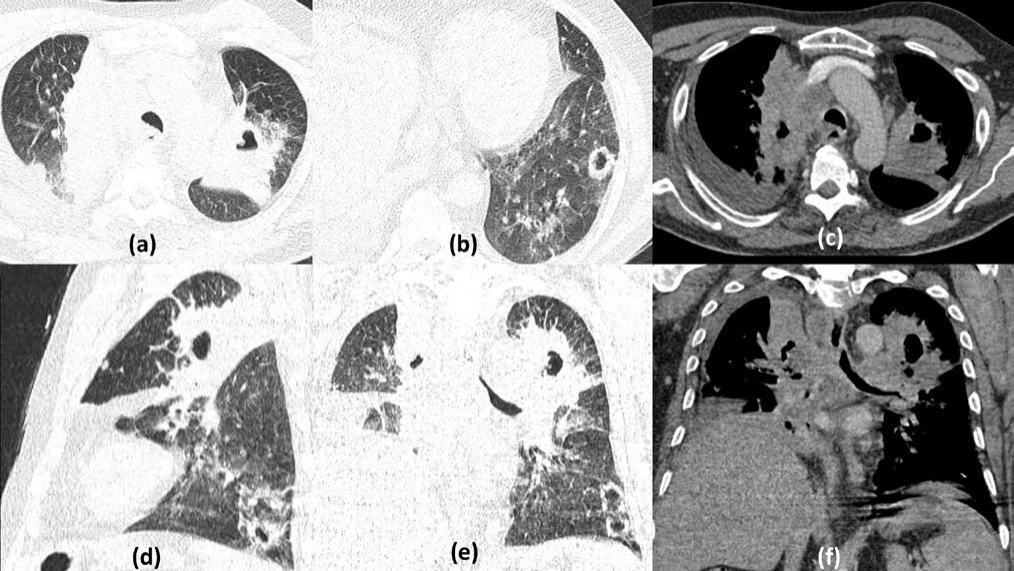
Primary lung cancer with multiple cavitating nodules and COVID19 infection: 60-year-old smoker with hemoptysis, shortness of breath and fever tested positive for COVID19 virus. Chest CT sections show a large mass lesion in the left parahilar region and upper lobe showing areas of central cavitation having irregular inner walls with spiculated outer margins, with extension into the fissure (sagittal section d). Multiple cavitating nodules are seen the lower segments. Focal areas of ground glass opacities are seen in the left lower lobe consistent with COVID19 (zoomed axial image b). On the contrast enhanced sections (axial c and coronal f) the mass demonstrates heterogenous enhancement. Also seen are multiple bulky necrotic mediastinal lymph nodes causing collapse of contralateral upper lobe and right-sided pleural effusion.

Cavitation can be seen in primary lung cancers in up to 20 percent of the cases with squamous cell being the most common histological type associated with it, followed by adenocarcinoma. On imaging, the cavity typically shows thick irregular walls with a thickness of more than 15 mm associated with a greater likelihood of malignancy^16^(Figure 13). Cavitation may also occur in a lung mass secondary to treatment with novel chemotherapeutic agents (like anti angiogenic factors)^17^ as well as with radiation therapy, due to central necrosis of the tumor.

#### Pulmonary metastasis

**Figure 14.**
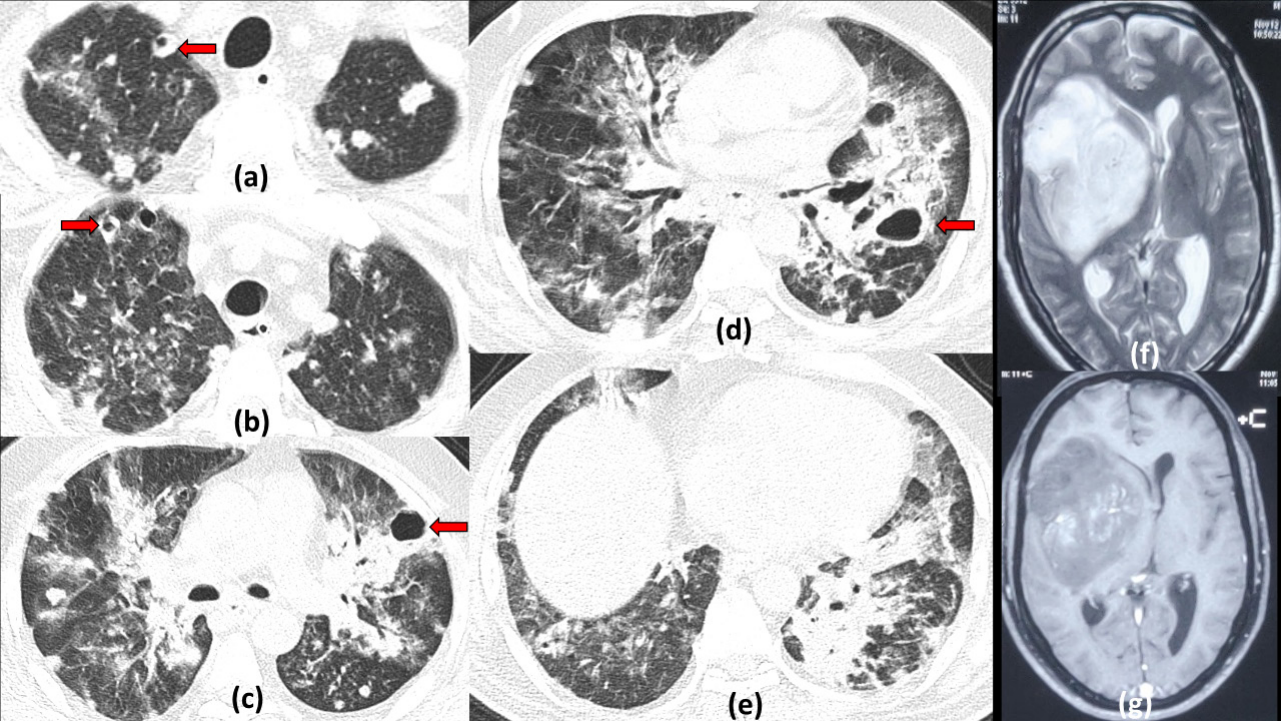
Cavitating pulmonary metastasis with COVID19 pneumonitis: 45-years-old male with known glioblastoma of right fronto-temporal lobes presented with acute onset dyspnoea and fever, Rapid antigen positive for COVID19. Axial chest HRCT sections (a–e) show multiple variable sized nodules dispersed peripherally in bilateral lung fields with few of them showing cavitation (red arrows). Multiple patchy ground glass opacities are also seen with areas of consolidation predominantly in the peripheral lower lobes reflecting COVID19 infective changes. In view of malignancy, CT-guided biopsy was done after patient became negative on RTPCR and cavitatory lesions were found to be metastasis. MRI brain axial *T*_2_ weighted image (f) and post contrast *T*_1_ weighted image (g) show a large lobulated mass lesion in the right fronto-temporal region showing patchy enhancement suggestive of primary lesion.

Pulmonary metastasis typically results in multiple variable sized peripherally located nodules, which can cavitate resulting in thick-walled irregular cavities. Metastasis from squamous cell carcinoma (most common), adenocarcinoma and sarcomas can show cavitation^16^ (Figure 14).

### Cavitation due to miscellaneous causes

**Figure 15.**
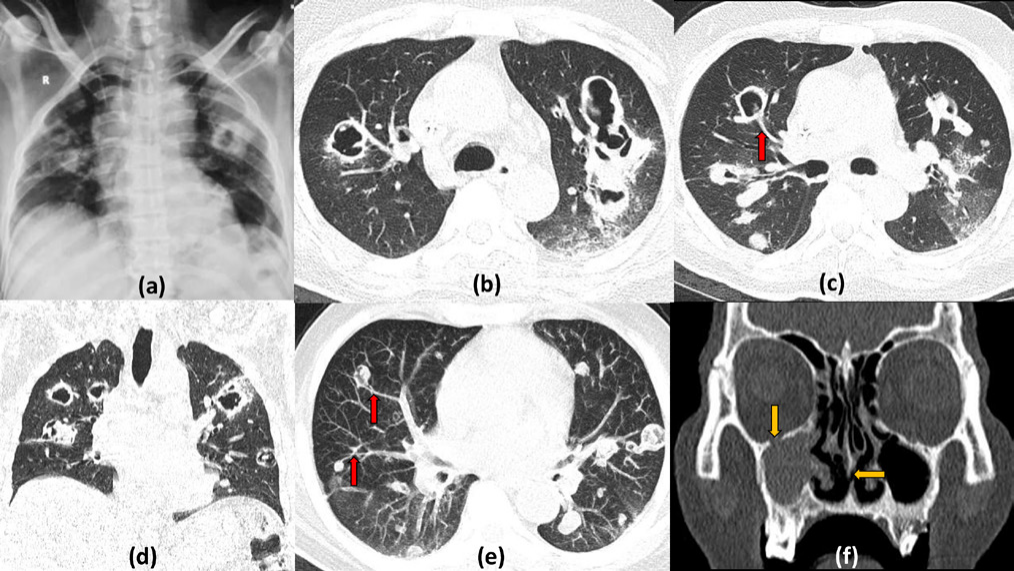
Multiple cavitating nodules of granulomatosis polyangiitis with COVID19 Pneumonitis: 55-year-old male presented to the emergency with acute shortness of breath and fever and tested positive for COVID19. Frontal radiograph shows evidence of multiple variable sized cavitating nodules in the bilateral lung fields. Chest CT lung window sections (b–e) show multiple well-defined nodules of varying sizes, most of which show central cavitation with *feeding vessel sign* seen (red arrows) better on the Maximum Intensity Projection image (e). Few focal ground glassing is seen in the posterior and basal areas of bilateral lung fields. Coronal CT of the paranasal sinuses, bone window (f) shows opacification of the right maxillary sinus with focal destruction of the nasal septum and erosion of the right orbital floor (yellow arrows).

Pulmonary nodules showing cavitation can be seen in Granulomatosis with polyangiitis (Wegener’s granulomatosis) - a granulomatous vasculitis which presents as multiple variable sized, peripheral predominant nodules showing cavitation, with often a vessel leading up to it that may be thrombosed.^15^ It can be associated with upper respiratory tract and multisystemic manifestations, frequently involving the kidneys (Figure 15). Rheumatoid arthritis (RA) can frequently have lung manifestations in the form of interstitial lung disease or rheumatoid nodules which may show cavitation.^16^

Nodular opacities due to embolism, metastasis, Wegener’s granulomatosis or RA may be difficult to differentiate from multifocal nodular opacities seen in about 20% cases of COVID19.^1^ However, the presence of cavitation should pre-empt a search for any primary malignancy, thrombus or septic foci, or other etiologies.

### Cystic lung lesions with coexistent COVID19 infection

#### Pneumothorax: mechanical ventilation associated barotrauma/Spontaneous

Pneumothorax and pneumomediastinum in COVID19 is often seen either due to mechanical ventilation associated barotrauma or spontaneously.^18^

**Figure 16.**
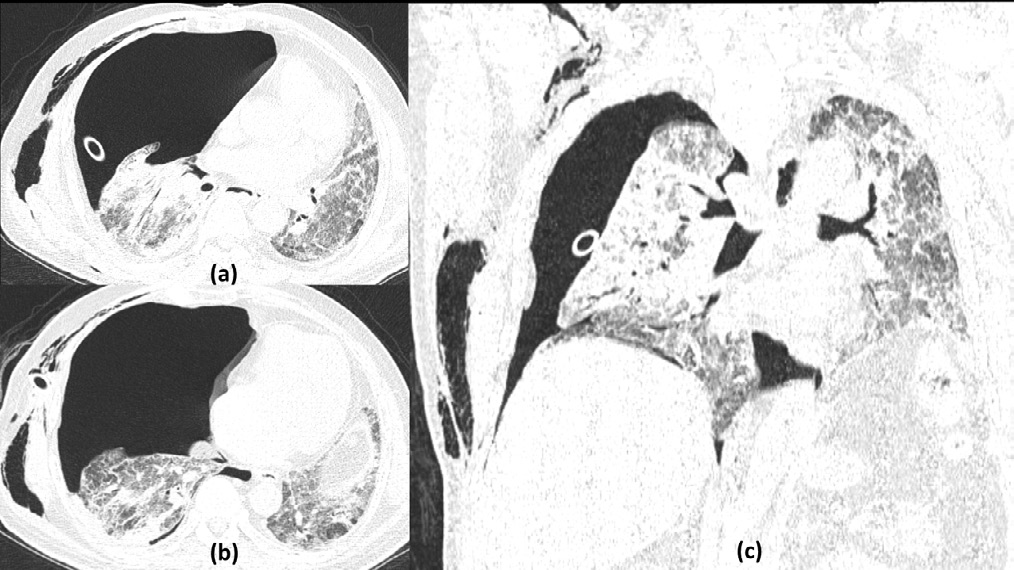
Pneumothorax with subcutaneous emphysema in severe COVID19 infection: 48-years-old male with severe COVID19 on non-invasive positive pressure ventilation. Axial and coronal chest HRCT images (a to c) reveal severe right-sided pneumothorax causing contralateral mediastinal shift with an Intercostal tube *in situ*, and subcutaneous emphysema. The underlying lung parenchyma shows diffuse ground glass opacities and patchy consolidation with associated interlobular septal thickening reflecting severe COVID19 Pneumonitis.

On imaging, a contained pneumothorax can mimic a cavitatory lesion, appearing as a well-defined lucency without bronchovascular markings. However, compression of surrounding lung parenchyma and displaced visceral pleural line help in differentiation and suggest pneumothorax (Figure 16).

### Cystic bronchiectasis

Bronchiectasis can occur due to a variety of conditions including - sequelae of COVID19 related fibrosis, previous infections including mycobacterium tuberculosis, cystic fibrosis and various ciliary dyskinesias.^15^

**Figure 17.**
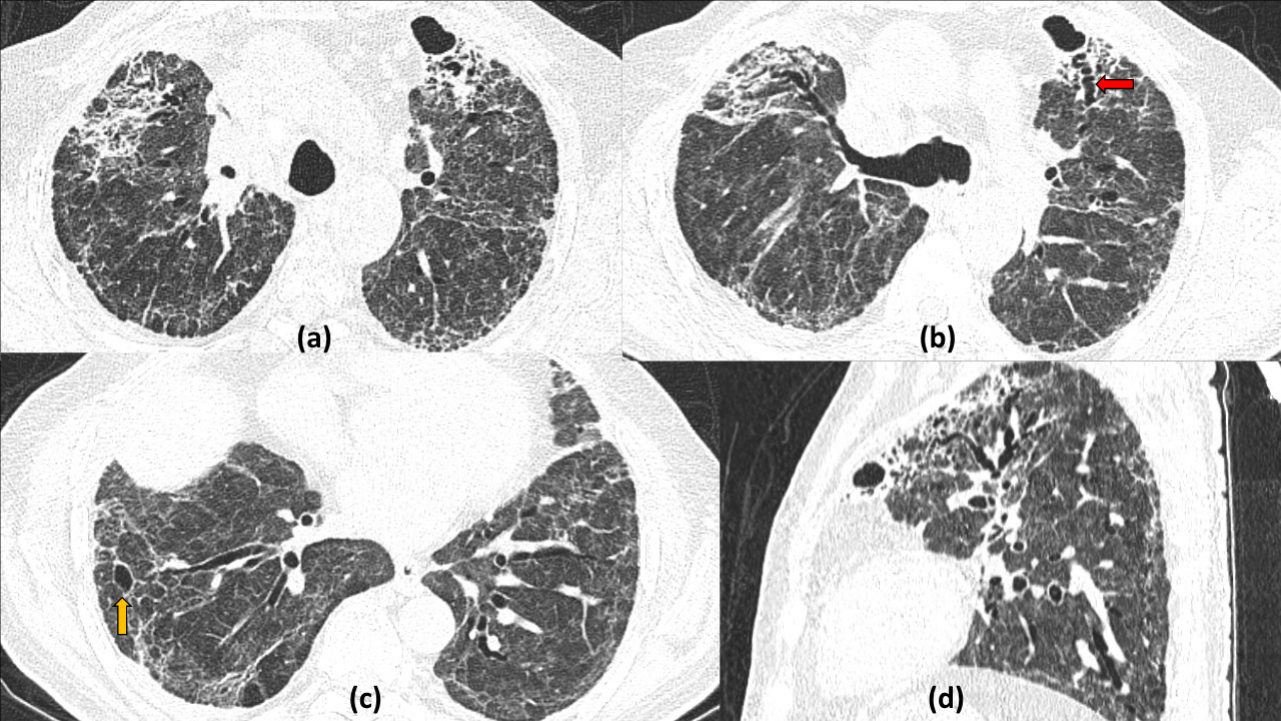
Cystic bronchiectasis and air spaces in sequelae of COVID19 infection: A 61-year-old male with severe COVID19 infection 2 months back, now RTPCR negative with recurrent dyspnoea. Axial (a–c) and sagittal reformatted (**d**) Chest HRCT sections show bilateral global tubular bronchiectatic changes. An addition few small cystic lesions are seen in the anterior paraseptal region of left upper lobe which are in continuation with a dilated bronchus (red arrow in b) suggestive of cystic bronchiectasis. Another cystic air space is also seen in the lateral basal segment of right lower lobe (yellow arrow in c). Note is made of marked peripheral subpleural interlobular septal thickening, parenchymal bands, architectural distortion suggestive of post-COVID19 fibrotic changes.

**Figure 18.**
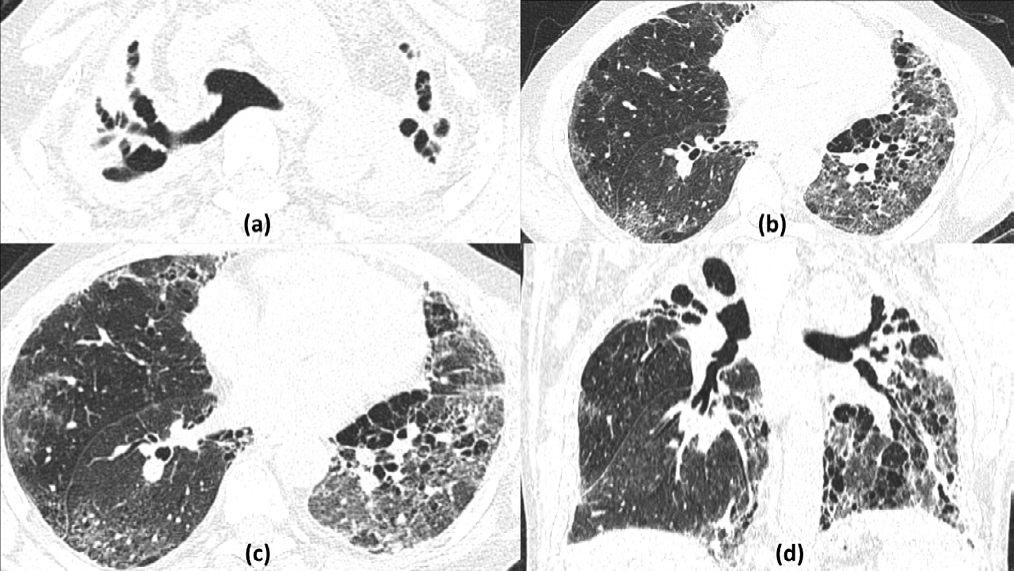
Cystic bronchiectasis due to previous *Mycobacterium tuberculosis* with COVID19 infection: 47-year-old male with past history of pulmonary tuberculosis, developed moderate to severe COVID19 pneumonitis. Chest HRCT (axial and coronal sections a –d) done on Day 23 of illness revealed severe cystic bronchiectasis predominantly in bilateral upper lobes with volume loss likely due to previous tuberculosis. Multiple patchy peripheral ground glass opacities with interlobular septal thickening and parenchymal bands are also seen suggesting post-COVID fibrotic sequelae.

**Figure 19.**
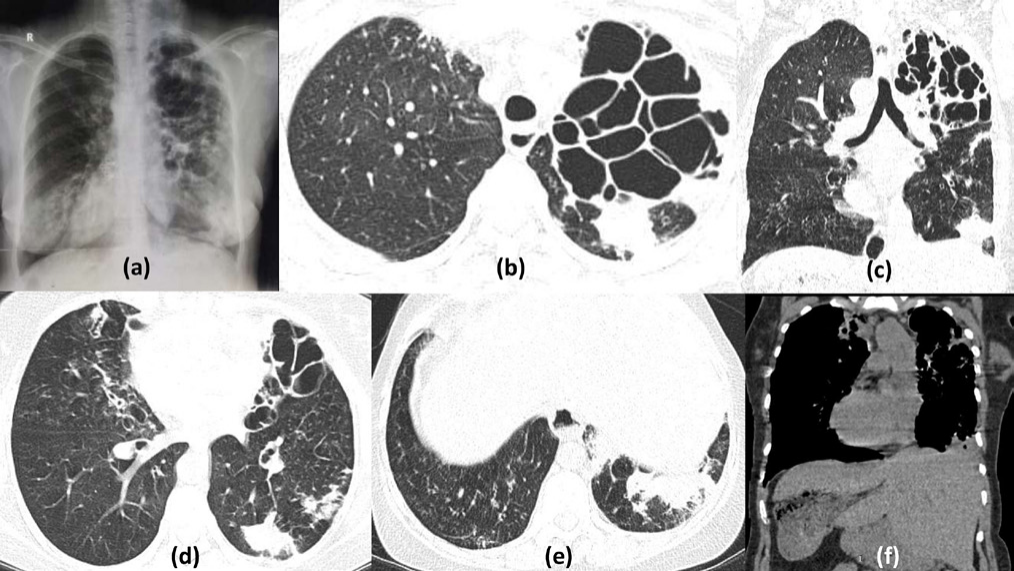
Cystic bronchiectasis with COVID19 infection in Kartagener Syndrome: 26-year-old female with chronic sinusitis and low-grade fever with cough, tested positive for COVID19 infection. Frontal radiograph (a) shows bilateral asymmetrical bronchiectasis with patchy peripheral air space opacities in the left lower zone. Note is made of situs inversus. Chest HRCT done on Day 20 of illness, axial sections (b, d and e) show patchy peripheral consolidations with extensive cystic bronchiectasis involving the entire left upper lobe and right middle lobe. Coronal lung (c) and mediastinal window (f) sections reveal bilateral hyparterial bronchus, dextrocardia, left-sided liver with right-sided stomach suggestive of complete situs inversus.

**Figure 20.**
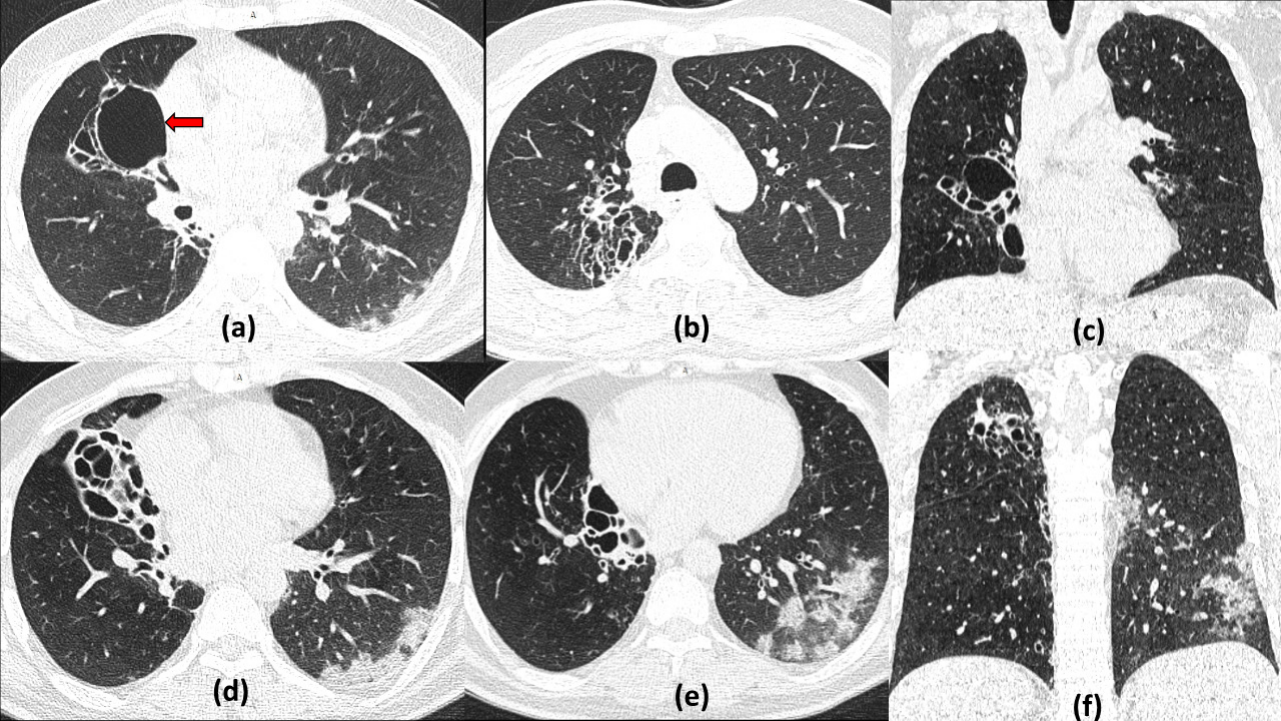
Cystic bronchiectasis with COVID19 infection mimicking cavities: 48-year-old male with history of previous lower respiratory tract infection and now RTPCR positive for COVID19 infection. Axial and coronal chest HRCT sections on Day 18 of illness show a large cystic lesion with thick walls in the right middle lobe mimicking a cavity (arrow in a). However, on contiguous sections (d) it was observed to be communicating with the trachea-bronchial tree suggestive of cystic bronchiectasis, which was also seen involving the posterior segment of right upper lobe. Multiple patchy peripheral subpleural consolidations are seen in the left lower lobe suggestive of COVID19 pneumonitis.

The distribution (craniocaudal, peripheral or central) and associated findings help to point to the cause of bronchiectasis. Tractional bronchiectasis at the sites of previous consolidation or ground glass opacities with other signs of fibrosis in a patient with history of previous or current COVID19 infection can suggest post-COVID19 bronchiectasis, whereas asymmetrical upper lobe bronchiectasis with volume loss and calcified granulomas in an endemic nation can be suggestive of post-tubercular bronchiectasis (Figures 17–20).

### Emphysematous bullae

Emphysematous bullae can be encountered in a COVID19 positive patient on chest CT and can mimic cystic air spaces associated with COVID19 pneumonia. Bulla appears as an air lucency (>1 cm size) with thin imperceptible walls^15^ usually having an apical or subpleural, as well as a centrilobular location, with other features of emphysema like low attenuation areas and vascular pruning. Cystic air spaces associated with COVID19 on the other hand, have a non-random distribution and are usually confined to areas of COVID19 pneumonitis (Figure 6).

### Solitary cyst or pneumatocoele

**Figure 21.**
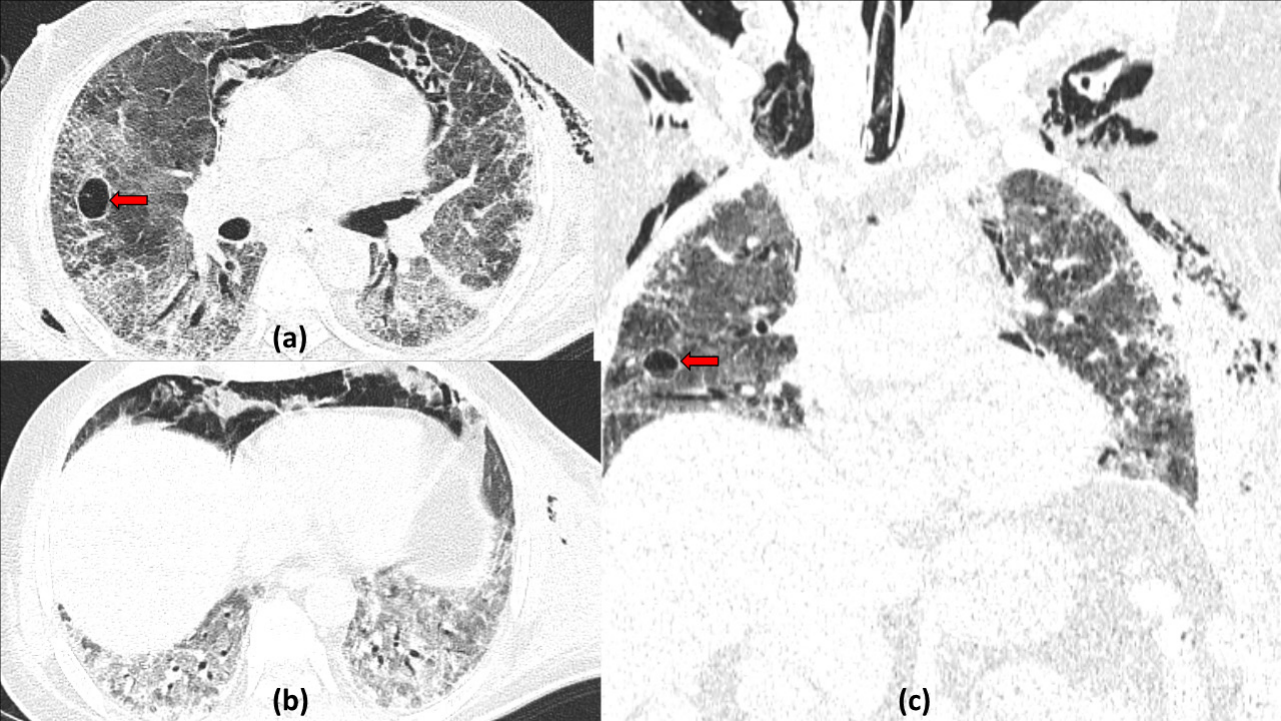
Simple cyst/pneumatocele with pneumomediastinum and subcutaneous emphysema in severe COVID19 pneumonitis: 53-year-old male with severe COVID19 infection and persistently low saturation necessitating invasive mechanical ventilation. Axial (a, b) and coronal (c) lung window sections on Day 21 of illness show a well-defined oval to round thin-walled simple cyst (red arrow) in the anterior segment of right upper lobe in a background of diffuse ground glass opacities and interlobular septal thickening. Note is made of endotracheal tube *in situ* with associated pneumomediastinum and subcutaneous emphysema suggesting barotrauma associated changes.

Lung cysts or pneumatocoeles have been reported to be associated with COVID19 infection in areas of ground glass opacities.^11^ These are often solitary and larger unlike cystic air spaces which are multiple and smaller (Figure 21).

### Honeycombing - usual interstitial pneumonia

**Figure 22.**
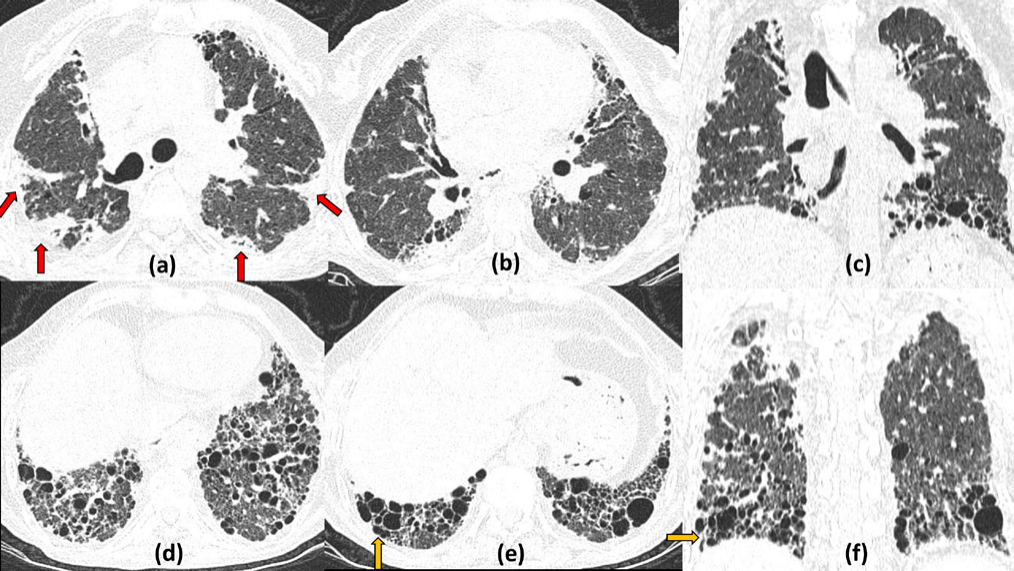
Usual Interstitial Pneumonia with COVID19 infection: 45-year-old female, a known case of UIP was diagnosed with COVID19 infection. Chest HRCT axial sections (a, b, d, e) show peripheral patchy consolidations predominantly in the posterior segments (red arrows). Also seen in marked bilateral basal predominant honeycombing (yellow arrows) and interlobular septal thickening. Coronal reformatted sections (c and f) demonstrate the basal gradient of the honeycombing (characteristic of UIP) and the peripheral patchy consolidations due to COVID19 pneumonitis. UIP, usual interstitial pneumonia.

Usual interstitial pneumonia (UIP) can result in pulmonary fibrosis causing multiple thin-walled cysts stacked in contiguous rows predominantly in the subpleural, posterior and bilateral basal locations^15^ with associated features of fibrosis like interlobular septal thickening, fibrotic ground glass opacities and volume loss. This typical distribution and overlap of imaging features can be seen in the late stage of COVID19 infection making diagnosis difficult, however, an acute history and absence of honeycombing favors a diagnosis of post-COVID19 fibrosis (Figure 22).

### Complicated cysts

Various lesions like hydatid cyst and intrapulmonary bronchogenic cyst may show internal air foci due to complications like secondary infection or communication with the airway^16^ and can mimic cavitatory lesions. They are often solitary and relatively larger in size, in an otherwise normal lung parenchyma unlike most other etiologies.

## Conclusion

Although uncommon, cavitation and cystic air spaces can be seen with COVID19 infection and can have significant clinical and prognostic implications. These could be early pointers to other ominous etiologies coexisting with COVID19 or reflect the end stage of the disease itself. Awareness about their etiopathogenesis and imaging features can aid in honing the skills of the radiologist with a resultant prompt diagnosis and timely management.
